# Supplementary material for: Atomistic simulation of voltage activation of a truncated BK channel
Source: eLife. 2025 Sep 8;14:RP105895. doi: 10.7554/eLife.105895 (PMC12416887; doi:10.7554/eLife.105895)
Supplement: Supplementary file 1. [file elife-105895-supp1.docx]

## **Supplementary Information**

## **Atomistic Simulation of Voltage Activation of a Truncated BK Channel**

##

Zhiguang Jia, and Jianhan Chen^*^

Department of Chemistry, University of Massachusetts, Amherst, MA 01003, USA

* Corresponding Authors: Phone: 413-545-3386; Emails: [jianhanc@umass.edu](mailto:jianhanc@umass.edu)

**Summary of simulations.** The closed state structure is the fully equilibrated conformation derived from the Ca^2+^-free Cryo-EM structures of the *ac*BK channel (PDB 5tji), and the activated state structure (*sim 5* and *6*) was taken from the last snapshot of *sim2b*. The P-loop/filter (T273 to D292) and C-terminus were harmonically restrained with a force constant of 0.5 kcal.mol^-1^.Å^-2^ in all production simulations. The 7 VSD charged residues examined in *sims 3-6* are D153, R167, D186, R207, R210, R213 and E219.

| **Runs** | **Biased Potential** | **Initial State** | **Voltage (mV)** | **Platform** | **Simulation Lengths** |
| --- | --- | --- | --- | --- | --- |
| *sim 1* | N/A | closed | 0 | Anton 2 | 2.0 μs |
| *sim 2* | N/A | closed | 750 | Anton 2 | 10 μs x 2 (a, b) |
| *sim 3* | N/A | resting | 0 | GPU  Gromacs | 7 residues  x 11 *λ* * 4 ns |
| *sim 4* | N/A | resting | 750 | GPU  Gromacs | 7 residues  x 11 *λ* x 4 ns |
| *sim 5* | N/A | activated | 0 | GPU  Gromacs | 7 residues  x 11 *λ* x 4 ns |
| *sim 6* | N/A | activated | 750 | GPU  Gromacs | 7 residues  x 11 *λ* x 4 ns |
| *sim 7* | N/A | Cryo-EM open state | 750 | GPU  Gromacs | 0.4 μs x 1 |
| *sim 8* | SMD | closed | 0 | GPU  Gromacs | 0.8 μs x 2* |
| *sim 9* | N/A | activated | 300 | GPU  Gromacs | 1.0 μs x 3 |

* In *Sim 8*, the S5 and S6 became detached in two out of four replicas and the simulations were terminated before reaching the end of 0.8 μs.
